# Letter to the Editor: Adverse Reactions of Artificial Bone Graft Substitutes: Lessons Learned From Using Tricalcium Phosphate geneX^®^

**DOI:** 10.1007/s11999-013-3420-x

**Published:** 2013-12-10

**Authors:** Phillip Anthony Laycock, John J. Cooper

**Affiliations:** Product Development, Biocomposites Ltd., Keele Science Park, Keele, Staffordshire ST5 5NL UK

*To the editor:*

The paper by Friesenbichler and colleagues [2] reported the findings of a truncated clinical study involving 31 patients treated for bone tumors by curettage, and filling of the defect using geneX^®^ (Biocomposites Ltd., Staffordshire, UK).

“The reconstructive approach after intralesional curettage is controversial and clinical practice varies,” the authors reported. “Polymethylmethacrylate (PMMA), allografts or autografts have been used in several studies and complication rates up to 33% have been reported” [2].

The authors reported the use of geneX^®^ in 31 patients, of which 26 patients had no complications. Based on these figures alone, these data represent a 16% complication rate — less than the 33% quoted above when using allografts and autografts.

Two patients (Patients 21 and 30) developed a postoperative soft tissue cyst at 52 and 55 days respectively, which reduced in size within 2 months without surgical intervention. One of these patients (Patient 30) also received an allograft and a Vitoss^®^ Bone Graft Substitute from Stryker Orthopaedics (Mahwah, NJ, USA) — a fact not reported in this article. In accordance with the medical device regulations, an investigation and visit by our regulatory department was carried out. This visit revealed the concurrent use of Vitoss^®^ in one of the cases.

Three patients (Patients 2, 25, and 31) had postoperative aseptic inflammation. One of the patients, a 14-year-old juvenile identified as a worst case, also received allograft. This patient required revision surgery 1 month after surgery, and presented with severe damage to the skin. Histology showed a chronic, lymphocytic inflammation with plasma cells. It is questionable to attribute these adverse reactions to the presence of geneX^®^ alone considering the concurrent use of allograft.

The authors believed one of the reasons for the inflammation and soft tissue cyst formation was the overfilled and pressurized defect site when using allografts. The Instructions For Use for geneX^®^ advise against overfilling or pressurizing the treatment site, and also recommend not adding other substances to the product.

To conclude that this type of bone substitute should not be used in the treatment of bony defects as a result of these data would appear presumptuous, and not warranted on the strength of the outcomes.

The comments made regarding the Saadoun paper [3] would suggest that the authors did not read the published response [1] or the clarification statement subsequently published by the authors [4]. geneX^®^ is cleared as a Class II medical device by the FDA, CE marked in accordance with the Medical Devices Directive 93/42/EEC, and complies with all safety and biocompatibility requirements. Since its launch in 2004, more than 20,000 packs of geneX^®^ have been used worldwide, and fewer than 0.24% reported complaints, according to our internal records (Procedure QAP0018).

geneX^®^ is safe and an effective bone void filler when used in accordance with the Instructions For Use.

## References

[1] Fitzer S, Cooper JJ (2011). Biocomposites Ltd response. J Neurol Neurosurg Psychiatry..

[2] Friesenbichler J, Maurer-Ertl W, Sadoghi P, Pirker-Fruehauf U, Bodo K, Leithner A. Adverse Reactions of Artificial Bone Graft Substitutes: Lessons Learned From Using Tricalcium Phosphate geneX^®^. [Published online ahead of print September 28, 2013]. *Clin Orthop Relat Res*. DOI: 10.1007/s11999-013-3305-z.

[3] Saadoun S, Macdonald C, Bell BA, Papadopoulos MC (2011). Dangers of bone graft substitutes: lessons from using geneX^®^. J Neurol Neurosurg Psychiatry..

[4] Saadoun S, Macdonald C, Bell BA, Papadopoulos MC (2011). Dangers of bone graft substitutes: lessons from using geneX^®^. Clarification. J Neurol Neurosurg Psychiatry..

